# Pemetrexed disodium in recurrent locally advanced or metastatic squamous cell carcinoma of the head and neck

**DOI:** 10.1054/bjoc.2001.2010

**Published:** 2001-09

**Authors:** X Pivot, E Raymond, B Laguerre, M Degardin, L Cals, J P Armand, J L Lefebvre, D Gedouin, V Ripoche, L Kayitalire, C Niyikiza, R Johnson, J Latz, M Schneider

**Affiliations:** Centre Antoine Lacassagne, Nice, France; Institut Gustave Roussy, Villejuif, France; Centre Eugène Marquis, Rennes, France; Institut Oscar Lambret, Lille, France; Hopital Font-pré, Toulon, France; Eli Lilly and Company, SA, France; Eli Lilly and Company, Indianapolis, IN, USA

**Keywords:** LY231514, pemetrexed disodium, ALIMTA®, multitargeted antifolate, head and neck squamous cell carcinoma, chemotherapy

## Abstract

This phase II study determined response rate of patients with locally advanced or metastatic head and neck cancer treated with pemetrexed disodium, a new multitargeted antifolate that inhibits thymidylate synthase, dihydrofolate reductase and glycinamide ribonucleotide formyl transferase. 35 patients with local or metastatic relapse of squamous cell carcinoma of the head and neck (31 male, 4 female; median age 53 years) were treated with pemetrexed 500 mg m^2^ administered as a 10-minute infusion on day 1 of a 21-day cycle. Patients received 1 to 8 cycles of therapy. 9 patients (26.5%) had an objective response, with a median response duration of 5.6 months (range 2.9–20 months). 15 (44.1%) had stable disease, and 8 (23.5%) had progressive disease. 2 patients were not assessable for response. Median overall survival was 6.4 months (range 0.7–28.1 months; 95% CI: 3.9–7.7 months). 24 patients (68.6%) experienced grade 3/4 neutropenia, with febrile neutropenia in 4 (11.4%). Grade 3/4 anaemia and thrombocytopenia occurred in 11 (34.3%) and 6 (17.1%) patients, respectively. The most frequent non-haematological toxicity was grade 3/4 mucositis (17.1%; 6 patients). In conclusion, pemetrexed is active in squamous cell carcinoma of the head and neck. Although substantial haematological toxicities were experienced by patients, subsequent studies have shown that these toxicities can be proactively managed by folic acid and vitamin B_12_ supplementation. © 2001 Cancer Research Campaign http://www.bjcancer.com


British Journal of Cancer (2001) **85**(5), 649–655
© 2001 Cancer Research Campaign
doi: 10.1054/ bjoc.2001.2010, available online at http://www.idealibrary.com on IDEAL
http://www.bjcancer.com

### Pemetrexed disodium in recurrent locally advanced or metastatic squamous cell carcinoma of the head and neck

**X Pivot[1], E Raymond[2], B Laguerre[3], M Degardin[4], L Cals[5], JP Armand[2], JL Lefebvre[4], D Gedouin[3], V Ripoche[6], L Kayitalire[6], C Niyikiza[7], R Johnson[7], J Latz and M Schneider[1]**

[1]Centre Antoine Lacassagne, Nice; France; [2]Institut Gustave Roussy, Villejuif, France; [3]Centre Eugène Marquis, Rennes, France; [4]Institut Oscar Lambret, Lille, France; [5]Hopital Font-pré, Toulon, France; [6]Eli Lilly and Company, SA, France, [7]Eli Lilly and Company, Indianapolis, IN, USA

**Summary** This phase II study determined response rate of patients with locally advanced or metastatic head and neck cancer treated with pemetrexed disodium, a new multitargeted antifolate that inhibits thymidylate synthase, dihydrofolate reductase and glycinamide ribonucleotide formyl transferase. 35 patients with local or metastatic relapse of squamous cell carcinoma of the head and neck (31 male, 4 female; median age 53 years) were treated with pemetrexed 500 mg $m^2$ administered as a 10-minute infusion on day 1 of a 21-day cycle. Patients received 1 to 8 cycles of therapy. 9 patients (26.5%) had an objective response, with a median response duration of 5.6 months (range 2.9–20 months). 15 (44.1%) had stable disease, and 8 (23.5%) had progressive disease. 2 patients were not assessable for response. Median overall survival was 6.4 months (range 0.7–28.1 months; 95% CI: 3.9–7.7 months). 24 patients (68.6%) experienced grade 3/4 neutropenia, with febrile neutropenia in 4 (11.4%). Grade 3/4 anaemia and thrombocytopenia occurred in 11 (34.3%) and 6 (17.1%) patients, respectively. The most frequent non-haematological toxicity was grade 3/4 mucositis (17.1%; 6 patients). In conclusion, pemetrexed is active in squamous cell carcinoma of the head and neck. Although substantial haematological toxicities were experienced by patients, subsequent studies have shown that these toxicities can be proactively managed by folic acid and vitamin $B_{12}$ supplementation.
© 2001 Cancer Research Campaign http://www.bjcancer.com

**Keywords**: LY231514; pemetrexed disodium; ALIMTA®; multitargeted antifolate; head and neck squamous cell carcinoma; chemotherapy

Squamous cell carcinoma of the head and neck (SCCHN) is the sixth most common form of cancer worldwide, with more than 500 000 new cases occurring in 1999 (Landis et al, 1999). In the United Kingdom, 6650 new cases and 2000 deaths from this disease were reported in 1997 (World Health Organization, 2000). Patients who present with an early stage of SCCHN can be successfully treated with a combination of surgery and/or radiation. However, up to 75% of SCCHN patients develop advanced locoregional disease (T2–T4, N2–N3) (Vokes et al, 1993). For these patients, the clinical outcome with traditional surgery and/or radiation is much less satisfactory. Newer therapies have led to alternative treatment regimens for these patients. Induction chemotherapy has led to organ preservation via a reduction of surgical indication, and chemoradiotherapy has improved survival in patients with inoperable disease (Dimery et al, 1993). Patients with advanced disease at diagnosis have a 5-year survival rate of only 30% and approximately 6 in 10 patients will experience a locoregional relapse within 2 years of initial treatment. The prognosis for patients with locally recurrent or metastatic disease is dismal, with a median survival time of approximately 6 months (Pivot et al, 2000). However, salvage options, such as additional surgery or radiation therapy, are limited in these cases. Recurrent locoregional disease is generally incurable, as is metastatic disease that occurs in approximately 20% of patients. For these patients, chemotherapy has become commonly accepted as standard therapy and used primarily as a palliative treatment.

*Received 28 November 2000*
*Revised 20 April 2001*
*Accepted 4 July 2001*

*Correspondence to:* Xavier Pivot

Methotrexate monotherapy is considered to be a standard therapy for recurrent head and neck cancer. Tumour response rates to single-agent methotrexate therapy range from 10% to 17%, with a median response duration of approximately 3 months (Schornagel et al, 1995). Survival for this patient population has not changed appreciably in the past 2 decades. While combination drug regimens have produced encouraging response rates, significantly higher than those observed with single-agent methotrexate alone, no significant survival advantage has been demonstrated (Vogl et al, 1985; Anon, 1990; Forastiere et al, 1992). Investigation of new therapies is needed in order to improve overall survival for these patients.

Pemetrexed disodium is a structurally modified folate analogue that suppresses both DNA synthesis and folate metabolism. Specifically, pemetrexed potently inhibits thymidylate synthase (TS) and dihydrofolate reductase (DHFR). This antimetabolite has also been found to inhibit glycinamide ribonucleotide formyl transferase (GARFT) and aminoimidazole carboxamide ribonucleotide formyltransferase (Shih et al, 1997; Chen et al, 1999). Pemetrexed is a classic antifolate that is converted intracellularly to polyglutamated derivatives by folypolyglutamate synthase. These polyglutamated forms have shown much greater inhibitory activity against TS and GARFT than its parent compound, although the inhibition of DHFR was unaffected by polyglutamation (Mendelsohn et al, 1999).

BJOC 01-2010 649-655 20/8/01 3:57 pm Page 650

Pemetrexed has demonstrated single-agent activity in a wide range of tumours, including breast, non-small-cell lung, colorectal and pancreas cancers (O'Dwyer, 1999; Postmus and Green, 1999; Rusthoven et al, 1999; John et al, 2000). Response rates have ranged from 31% in breast and 16–23% in non-small-cell lung cancers to 16% in colorectal and 6% in pancreatic cancers. Phase II studies thus far have employed a dose schedule of pemetrexed disodium at either 500 or 600 mg $m^{-2}$ as a 10-minute i.v. infusion once every 21 days. As expected from a DNA synthesis inhibitor, the most serious and common adverse events were haematological toxicities. Because of the broad range of activity of pemetrexed disodium in various cancers, the present study was undertaken to investigate the safety and efficacy of this agent in patients with locally advanced or metastatic head and neck cancers. Additionally, the study assessed the pharmacodynamics and pharmacokinetics of pemetrexed disodium in this patient population.

### MATERIALS AND METHODS

### Patient eligibility

Patients were eligible for this trial if they had histologically confirmed squamous cell carcinoma of the head and neck that recurred locally or as distant metastatic disease. A bidimensionally measurable lesion with clearly defined margins was required. Lesions could not be amenable to curative surgery or radiotherapy. Prior radiotherapy was allowed, but a minimum of 6 months was required between the completion of prior radiotherapy and entry into the study and the irradiated area could not be the only site of measurable disease. Prior chemotherapy was allowed only if used as an induction strategy or as a concomitant chemoradiotherapy treatment and must have been completed 6 months prior to study entry. An estimated life expectancy of at least 12 weeks and an Eastern Cooperative Oncology Group performance status of 0 to 2 were required for study entry. Adequate organ function as defined by the following was required: platelets $\geq 100 \times 10^9\ l^{-1}$, haemoglobin $\geq 9$ g $dl^{-1}$, absolute granulocyte count (AGC) $\geq 2.0 \times 10^9\ l^{-1}$, bilirubin $\leq 1.5$ times the upper limit of normal, aspartate transaminase (AST) or alanine transaminase (ALT) $\leq 3.0$ times normal (AST or ALT $\leq 5$ times normal was acceptable if due to liver disease), and calculated creatinine clearance $\geq$ 45 ml $min^{-1}$. Patients had to give informed consent.

Patients were excluded from the study if they had a second primary malignancy, except for an in situ carcinoma of the cervix or adequately treated basal cell carcinoma of the skin or other malignancy treated at least 5 years previously with no evidence of recurrence. Patients with clinically significant effusions (pleural or peritoneal) or albumin < 2.5 gm $dl^{-1}$ were ineligible for the study. Additionally, patients could not have undifferentiated and non-keratinizing carcinomas. Patients with active infection or symptomatic brain metastasis or who were pregnant were excluded from the study. The use of any investigational agent 30 days before enrollment in the study was forbidden. Patients who could not discontinue therapy with aspirin and other non-steroidal anti-inflammatory agents for 2 days before, the day of, and 2 days after administration of pemetrexed disodium were excluded from the trial.

### Study design

This phase II multicentre study was designed as a single-arm, open-label, two-stage, (Fleming et al, 1997) nonrandomized trial. Pemetrexed was administered at a dose of 500 mg $m^{-2}$ as a 10-minute i.v. infusion once every 21 days. Dexamethasone 4 mg, a prophylactic measure for skin rash, was taken orally twice a day starting the day before and continuing until the day after pemetrexed administration (Calvert and Walling, 1998). Dose adjustments were based on nadir counts and/or maximal non-haematological toxicity from the preceding cycle of therapy. Specifically, grade 3 and grade 4 mucositis required a 25% and a 50% dose reductions, respectively. Grade 4 neutropenia lasting more than 5 days required a 15% dose reduction. Grade 3 and grade 4 thrombocytopenia required 15% and 50% dose reductions, respectively. A 25% dose reduction was required in case of concomitant grade 4 neutropenia and grade 3 thrombocytopenia. Neutrophil count was required to be $>1.5 \times 10^9\ l^{-1}$ and platelets $> 100 \times 10^9\ l^{-1}$ prior to the start of any cycle. Otherwise, treatment was delayed up to 2 weeks to allow sufficient time for recovery. If, after the 2-week delay, neutrophil and/or platelet counts had not surpassed these levels, then the patient was removed from the study. If a patient had a calculated creatinine clearance < 45 ml $min^{-1}$, then the next cycle would not begin until the calculated creatinine clearance value was $\geq$ 45 ml $min^{-1}$. If a patient's calculated creatinine clearance had not returned to $\geq$ 45 ml $min^{-1}$ within 6 weeks, then the patient was removed from the study. No other chemotherapy, immunotherapy, hormonal cancer therapy (excluding contraceptives and corticosteroids), radiation therapy or experimental medications were allowed. Use of granulocyte colony stimulating factors was not allowed.

### Response assessment

Tumour measurements were assessed by radiological imaging within 4 weeks prior to study entry and every 6 weeks thereafter. Specifically, evaluation of efficacy was performed during the third week following the administration of cycles 2, 4 and 6. All responses were reviewed by a panel of independent investigators and were determined using SWOG criteria. A complete response (CR) was defined as the disappearance of all measurable and evaluable disease, with no new lesion. Partial response (PR) was applied to patients who had a decrease in the sum of products of all measurable lesions greater than or equal to 50%, with no progression of evaluable disease and no new lesions. Progressive disease (PD) was defined as a 25% increase in the sum of products of all measurable lesions, the reappearance of any lesion which had disappeared, or the appearance of any new lesion. Stable disease (SD) was defined as any response which did not qualify as a CR, PR or PD. All responding patients were required to have a 4-week confirmation imaging. The duration of response was defined as the time from first objective status assessment of CR or PR to the first sign of disease progression or death due to any cause. Time-to-treatment failure (TTF) was defined as the time from study entry to the first observation of disease progression or death due to any cause or early discontinuation of treatment. Overall survival (OS) was defined as the time from study entry to time of death due to any cause.

### Pharmacokinetic analysis

Blood samples were collected for analysis of pemetrexed in plasma during cycles 1 and 3. The samples were collected according to a randomization schedule during each of 4 collection intervals: 0 to 2, 2 to 6, 6 to 12 and 12 to 36 hours after the start of

 © 2001 Cancer Research Campaign infusion for each of these 2 cycles. These time windows were selected based on the full pharmacokinetic profile obtained from a phase I study (Rinaldi et al, 1999) and will provide an adequate characterization of the pharmacokinetic profile after intravenous administration.

The pharmacokinetics of pemetrexed in patients with head and neck cancer were evaluated as part of a population pharmacokinetic model developed from plasma pemetrexed concentration–time data collected in patients with colorectal, pancreatic, breast, oesophageal, renal, head and neck, bladder or cervical cancers. Plasma concentration–time data from 8 studies, each evaluating a single cancer type, were combined into a single data set and analysed. An open 2-compartment model parameterized in terms of clearances and volumes was used for the structural model.

Pharmacokinetic analysis was performed using population pharmacokinetic methods (Mandema et al, 1992) with the non-linear mixed-effects modelling program, NONMEM (Version 5, PREDPP 5). A final population pharmacokinetic model including statistically significant covariates was developed using a data set containing 1184 concentrations from 209 patients; of these, 162 concentrations were from 26 patients with SCCHN (analyses on file). Plasma clearance (CL) was dependent on creatinine clearance. Central volume of distribution ($V_1$) was a function of body surface area. Peripheral volume ($V_2$) and intercompartmental clearance (Q) were independent of the covariates examined.

The effect of the study population (i.e. cancer type) was evaluated by comparing typical CL values between the patients with SCCHN and those patients without SCCHN using the final population pharmacokinetic model and adding an additional parameter to the final model (Eq. 1) as follows:

$$CL_{SCCHN} = CL \cdot (1+\Theta_{SCCHN} \cdot IND) \quad (1)$$

where $CL_{SCCHN}$ is the plasma clearance in patients with head and neck cancer, CL is the plasma clearance from the population of patients that did not have head and neck cancer (Eq. 1.), $\Theta_{SCCHN}$ adjusts the plasma clearance in patients with SCCHN, and IND is a dichotomous indicator variable that distinguishes between the 2 populations. The numerical value of IND was 1 for patients with SCCHN and 0 for patients with the other cancer types.

Statistical inference concerning differences in CL between the 2 populations was based upon a comparison of minimum values of the NONMEM objective functions (MOF) between the final population model and the model incorporating an effect for study population on CL (Eq. 1). A decrease in the MOF of at least 3.841 (*P* value $\leq 0.05$; 1 degree of freedom) between the 2 models for CL is required for statistical significance.

### Statistical analysis

All estimated confidence interval parameters were designed with a significance level of $\alpha = 0.05$. The efficacy analysis was performed on data from all protocol-qualified patients. The safety analysis was performed on data from all patients who received at least one dose of the study drug. Time-to-event endpoints of overall survival (OS), time-to-failure (TTF), and response duration were evaluated with Kaplan–Meier non-parametric methods using JMP® Software (SAS, Cary NC). The pharmacodynamic and pharmacokinetic comparisons of the pemetrexed disodium blood concentration between patients from this study and other phase II studies were performed using a Spearman rank test.

### RESULTS

### Patient characteristics

Between May 1997 and February 1999, 35 patients (31 males, 4 females) were enrolled in the study. All patients were assessable for safety and 34 patients were assessable for response. The median age was 53 years (range 38–75 years). Median ECOG performance status was 1 (range 0–2). Before entering into the study, all but 2 patients had been previously treated with surgery and/or radiotherapy. The 2 previously untreated patients had distant metastases at initial presentation. 14 patients (40%) had previously received chemotherapy (cisplatin-fluorouracil combination), either as induction therapy or as concomitant chemoradiotherapy. The median interval between initial treatment and relapse was 11 months (range 6–80 months). 31 patients (88.6%) had metastatic disease. Involved sites included lung (31 patients), liver (7 patients), mediastinal (2 patients) and bone (1 patient). 4 patients (11.4%) had only locoregional disease, and all recurrences were outside an area previously treated by radiotherapy. 18 patients (51.4%) had both locoregional recurrence and metastatic disease. Table 1 summarizes patient characteristics.

### Response and survival results

Among the 35 enrolled patients, 34 were considered evaluable for response according to protocol. One patient was not evaluable for efficacy because he did not have a bidimensionally measurable lesion outside an area previously untreated by radiotherapy. This patient achieved a PR in the oral cavity lesion and a CR in the lung lesion, with a response duration longer than 16 months.

Confirmed PR was observed in 9 patients, giving a 26.5% response rate (95% CI: 12.9–44.4%). The median response duration was 3.9 months (range 2.9–20 months; 95% CI: 2.1–7.2 months). SD was observed in 15 patients (44.1%), and 8 patients

**Table 1** Patient characteristics

| Characteristic | No. of patients | % |
|---|---|---|
| Entered | 35 | 100 |
| Evaluable | | |
| Safety | 35 | 100 |
| Efficacy | 34 | 97.1 |
| Age | | |
| Median | 53 years | |
| Range | 38–75 years | |
| Gender | | |
| Male | 31 | 88.6 |
| Female | 4 | 11.4 |
| ECOG performance status | | |
| 0 | 9 | 25.7 |
| 1 | 22 | 62.9 |
| 2 | 4 | 11.4 |
| Prior therapy | | |
| Surgery | 23 | 65.7 |
| Radiotherapy | 27 | 77.1 |
| Chemotherapy | 14 | 40.0 |
| Site of recurrence | | |
| Local recurrence only | 4 | 11.4 |
| Metastatic | 31 | 88.6 |
| Metastatic sites | | |
| Lung | 31 | 88.6 |
| Liver | 7 | 20 |
| Mediastinum | 2 | 5.7 |
| Bone | 1 | 2.9 |

*© 2001 Cancer Research Campaign*

BJOC 01-2010 649-655 20/8/01 3:57 pm Page 652

**Table 2** Response rates

| | Enrolled patients (*n* = 35) | Efficacy evaluable patients (*n* = 34) |
|---|---|---|
| Partial response | 9 (25.7%; 95% CI 18.2–33.2%) | 9 (26.5%; 95% CI: 12.9–44.4%) |
| Stable disease | 15 (42.9%; 95% CI 31.6–48.4%) | 15 (44.1%; 95% CI: 27.2–62.1%) |
| Progressive disease | 8 (22.9%; 95% CI 18.2–33.2%) | 8 (23.5%; 95% CI: 10.7–41.2%) |
| Not done | 3 (8.6%) | 2 (5.8%) |

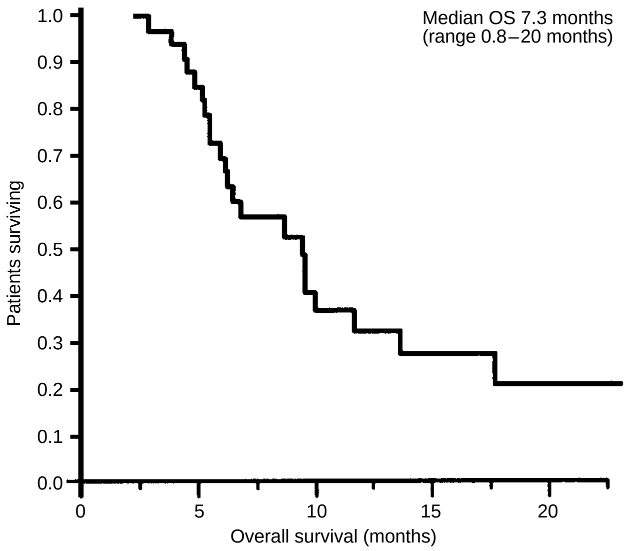

**Figure 1** Overall survival

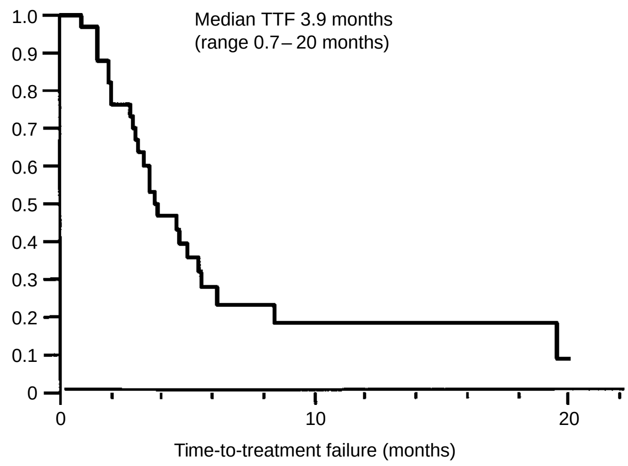

**Figure 2** Time-to-treatment failure

(23.5%) had a PD as a best response. In addition, 2 of the 34 efficacy-evaluable patients were not assessed for response. One patient died after one cycle of therapy due to haemorrhage possibly related to study drug. The second patient discontinued after one cycle of therapy due to infection possibly related to study drug. These results are detailed in Table 2.

At the time of this report, 5 of the 35 patients enrolled in the study are alive. Median overall survival for all patients was 7.3 months (range 0.7–28.1 months; 95% CI: 3.9–7.7 months) compared to 20.4 months (range 7.3–27.4 months; 95% CI: 7.3–28.1 months) for responders. Due to censoring, the upper end of the 95% confidence interval was nonestimable. The 1-year survival rate for all patients was estimated to be 29% (Figure 1). Median time-to-treatment failure was 3.9 months (range 0.7–28.1 months; 95% CI: 2.8–9.0 months) (Figure 2).

### Safety

24 patients (68.6%) experienced grade 3/4 neutropenia, with febrile neutropenia occurring in 4 patients (11.4%) (Table 3). Grade 3/4 anaemia and thrombocytopenia were observed in 12 (34.3%) and 6 (17.1%) patients, respectively. 2 patients died due to neutropenic sepsis, and 2 patients died due to tumoral bleeding associated with grade 4 thrombocytopenia. 2 patients discontinued the study after the first cycle due to pulmonary sepsis.

The most common grade 3/4 non-haematological toxicity was mucositis, observed in 6 patients (17.1%) (Table 4). Other severe non-haematologic toxicities occurred infrequently and included skin rash (3 patients; 8.6%), nausea (1 patient; 2.9%), vomiting (1 patient; 2.9%), and diarrhoea (1 patient; 2.9%).

A total of 134 cycles of pemetrexed was administered. The median number of cycles per patient was 4 (range 1–6 cycles). Grade 3/4 toxicities occurring by percent of cycle included neutropenia (35.8%), anaemia (10.5%), thrombocytopenia (5.2%), febrile neutropenia (5.2%), and mucositis (5.2%) (Table 5). The duration of neutropenia was short (median duration 1.9 days, ranged between 1 to 5 days), and there was no evidence of cumulative toxicity. Importantly, pemetrexed did not exhibit

**Table 3** Haematological toxicities (*n* = 35 patients)

| | Grade 0–1 | Grade 2 | Grade 3 | Grade 4 |
|---|---|---|---|---|
| Neutropenia | 5 (14.3%) | 6 (17.1%) | 10 (28.6%) | 14 (40.0%) |
| Leukopenia | 4 (11.4%) | 5 (14.3%) | 14 (40.0%) | 12 (34.3%) |
| Anaemia | 12 (34.3%) | 11 (31.4%) | 8 (22.9%) | 4 (11.4%) |
| Thrombocytopenia | 25 (71.5%) | 4 (11.4%) | 2 (5.7%) | 4 (11.4%) |
| Febrile neutropenia | | | | 4 (11.4%) |

**Table 4** Non-haematologic toxicities (*n* = 35 patients)

| | Grade 0–1 | Grade 2 | Grade 3 | Grade 4 |
|---|---|---|---|---|
| Mucositis | 22 (62.9%) | 7 (20.0%) | 4 (11.4%) | 2 (5.7%) |
| Skin rash | 27 (77.1%) | 5 (14.3%) | 3 (8.6%) | 0 |
| Nausea | 27 (77.1%) | 7 (20.0%) | 1 (2.9%) | 0 |
| Vomiting | 30 (85.7%) | 4 (11.4%) | 1 (2.9%) | 0 |
| Diarrhoea | 31 (88.5%) | 3 (8.6%) | 1 (2.9%) | 0 |
| Alopecia | 35 (100%) | 0 | 0 | 0 |

 © 2001 Cancer Research Campaign

BJOC 01-2010 649-655 20/8/01 3:57 pm Page 653

**Table 5** Toxicities per cycle ($n$ = 134 cycles)

| | Grade 0–1 | Grade 2 | Grade 3 | Grade 4 |
|---|---|---|---|---|
| Neutropenia | 63 (47.0%) | 23 (17.1%) | 25 (18.7%) | 23 (17.2%) |
| Leukopenia | 46 (34.3%) | 39 (29.1%) | 35 (26.1%) | 14 (10.5%) |
| Anaemia | 77 (57.5%) | 43 (32.1%) | 10 (7.5%) | 4 (3.0%) |
| Thrombocytopenia | 116 (86.6%) | 11 (8.2%) | 3 (2.2%) | 4 (3.0%) |
| Febrile neutropenia | | | | 7 (5.2%) |
| Mucositis | 120 (89.6%) | 7 (5.2%) | 6 (4.5%) | 1 (0.7%) |

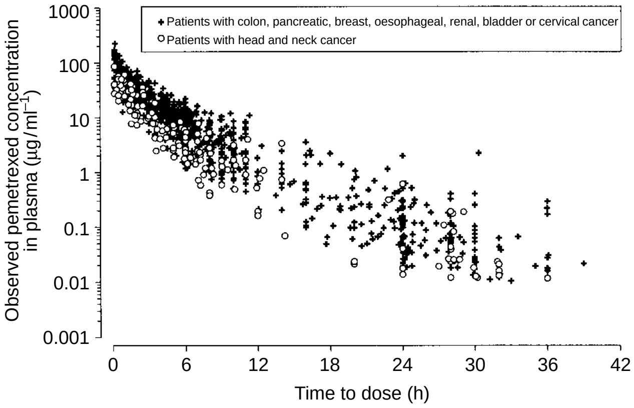

**Figure 3** Comparison of plasma pemmetrexed concentration versus time data

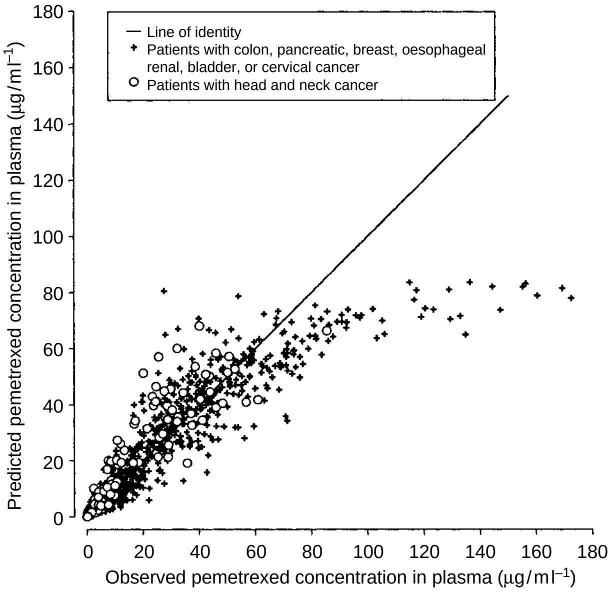

**Figure 4** Comparison of predicted and observed plasma pemetrexed concentrations in plasma

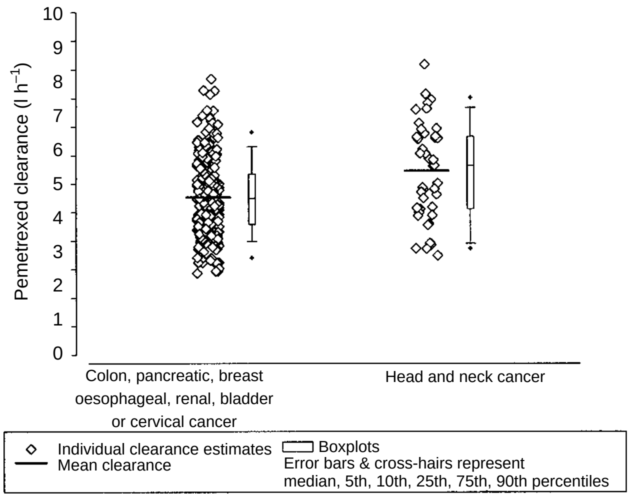

**Figure 5** Comparison off individual estimates of pemetrexed clearance significant renal toxicity. Transient deteriorations in liver function tests were observed, with grade 3 increases in serum levels of transaminases (15.7%) and bilirubin (4.5%), respectively; however, no clinical symptoms associated with these changes were noted.

86% of the planned dose intensity was delivered. 26% of patients required a dose reduction and 20% of patients had a 7-day delayed administration.

### Pharmacokinetic results

Figure 3 compares plasma pemetrexed concentration as a function of time for patients with SCCHN to those with other cancer types. Figure 4 shows the relationship between population predicted and observed plasma concentrations. Plasma concentrations from patients with SCCHN overlapped with those from patients with other cancer types. Therefore, systemic drug exposure in patients with SCCHN appeared to be consistent with that in patients with other cancer types.

The typical value of CL was estimated to be 8% higher ($\Theta_{SCCHN}$ = 0.08) in patients with SCCHN than in patients with other cancer types. This difference was not statistically significant since the MOF for the model incorporating the effect of study population was less than 3.841 points lower ($P$ value > 0.05) than the MOF for model that did not distinguish between SCCHN and other cancer types. Figure 5 illustrates the distribution of individual Bayesian estimates of CL between the 2 populations. Therefore, since systemic exposure in patients with SCCHN is consistent with that in patients with other cancer types, differences in safety results between the populations are not due to differences in pharmacokinetics.

### DISCUSSION

The results achieved with pemetrexed disodium in recurrent squamous cell carcinoma of the head and neck are encouraging. The 26.5% PR rate is one of the highest levels of activity for a single agent reported in this patient population. The 3.8-month response duration is similar to results obtained with single-agent or combination chemotherapy regimens for recurrent head and neck cancer (Jacobs et al, 1983; Vogl et al, 1985; Clavel et al, 1994; Schornagel et al, 1995). Likewise, the median overall survival of 6.4 months achieved in this trial was similar to that reported with other therapies.

© 2001 Cancer Research Campaign 

14 patients had previously received cisplatin-fluorouracil chemotherapy administered either as induction treatment or as concomitant chemoradiotherapy. Interestingly, of the 3 patients who had failed to respond to cisplatin-fluorouracil induction treatment, 2 patients achieved a PR with pemetrexed. This result suggests that pemetrexed disodium may be effective in patients with tumours resistant to cisplatin-fluorouracil therapy.

Several characteristics of the study population should be considered when analysing this data. Because eligibility criteria required the existence of at least one lesion outside an area previously treated by radiotherapy, the majority of patients (88.6%) in this study presented with distant metastases. Patients with metastatic head and neck cancer have poorer outcomes than those with recurrent locoregional disease (Pivot et al, 2000). Moreover, data suggest that chemotherapy may be more effective in locoregional relapse than in metastatic disease (Recondo et al, 1991). This difference in terms of activity between metastatic lesion and locoregional relapse was not consistent in all chemotherapy trials. A controversial study reported a higher response rate in patients with metastatic disease than for those with locoregional relapse (Shin et al, 1998). In a randomized trial, Schornagel et al (1995) reported that patients with locoregional disease achieved a higher response rate when compared to patients with metastatic disease (21% versus 13%, respectively). In the present study that included patients most likely to be least responsive to chemotherapy, the 26.5% response rate achieved with pemetrexed monotherapy is promising.

Although all phase II trials with pemetrexed include pharmacokinetic analysis, the objective of the pharmacokinetic analysis for this study was to determine if systemic drug exposure in patients with SCCHN differed from that in patients with other cancer types. As the trial progressed, it was evident that SCCHN patients were experiencing haematological toxicities higher than that previously observed in other pemetrexed trials. Two possible explanations emerged. First, nutritional status of SCCHN patients, which is generally poor, played a role in the incidence and severity of toxicities seen with this antifolate, or second, SCCHN patients were exposed to higher plasma concentrations of pemetrexed than patients in earlier studies. In order to investigate these possibilities, the pharmacokinetics of pemetrexed in SCCHN patients was compared to that in patients with other cancer types. The typical value of plasma clearance in patients with SCCHN was shown to be 8% higher than that from patients with other cancer types. However, this result was not statistically significant ($\Delta$MOF = –2.7; $P > 0.05$). Comparison of plasma concentrations and the distribution of individual CL values between the 2 populations showed a large degree of overlap, further illustrating the similarities in pemetrexed pharmacokinetics between the 2 populations. Since haematological toxicity of pemetrexed has been correlated with drug exposure, the increase in haematological toxicity in this study cannot be attributed to drug exposure alone and probably reflects the generally poor medical condition and advanced disease state of the patients in this study.

In support of this conclusion, early preclinical and clinical studies of antifolates have suggested a link between patient folate nutritional status and the likelihood of severe toxicity following treatment with an antifolate (Morgan et al, 1990; Smith et al, 1995; Alati et al, 1996; Mendelsohn et al, 1996). Niyikiza et al (1998) assessed the relationship between vitamin deficiency markers (homocysteine, cystathionine and methylmalonic acid), systemic pemetrexed exposure and patient toxicities. The authors observed that high homocysteine pretreatment levels, which reflect patient folate status, were closely related to an increased risk of grade 4 neutropenia and thrombocytopenia, as well as G3/4 diarrhoea and G3/4 mucositis. Head and neck cancer patients typically have poor nutritional status and this condition may play a role in the high incidence of haematological toxicity. Indeed following a programmatic change to supplement patients with folic acid and vitamin $B_{12}$, Bunn et al (2001) reported that haematological and non-haematological toxicities can be proactively managed.

In conclusion, pemetrexed has moderate activity in the treatment of patients with recurrent locally advanced or metastatic squamous cell carcinoma of the head and neck. Further investigation is warranted to evaluate the therapeutic efficacy of this new agent in terms of both tumour response and survival either as a single agent or in combination with other known active agents. Recently, routine addition of daily low-dose folic acid and vitamin $B_{12}$ has been instituted in studies involving pemetrexed disodium. This supplementation will likely reduce the toxicity in future studies, especially in nutritionally-challenged patients such as those studied in this investigation.

### ACKNOWLEDGEMENTS

The authors wish to thank Elaine Gorham for her support in preparing this manuscript.

### REFERENCES

Alati T, Worzalla JF, Shih C, Bewley JR, Lewis S, Moran RG and Grindey GB (1996) Augmentation of the therapeutic activity of lometrexol – (6-R) 5,10-dideazatetrahydrofolate- by oral folic acid. *Cancer Res* **56**: 2331–2335

Anon. (1990) A phase III randomised trial of cisplatinum, methotrextate, cisplatinum + methotrexate and cisplatinum + 5-FU in end stage squamous carcinoma of the head and neck. Liverpool Head and Neck Oncology Group. *Br J Cancer* **61**: 311–315 [erratum appears in (1990) *Br J Cancer*, **62**: 171]

Bunn P, Paoletti P, Niyikiza C, Rusthoven J, Nelson K, Hanauske A, Stabler S, Calvert H and Allen R (2001) Vitamin B12 and Folate reduce toxicity of Alimta (pemetrexed disodium, LY231514, MTA), a novel antifolate/antimetabolite. *Proceedings of ASCO* **A300** (Abstr.)

Calvert AH and Walling JM (1998) Clinical studies with MTA. *Br J Cancer* **78**: 35–40

Chen VJ, Bewley JR, Andis SL, Schultz RM, Iverson PW, Shih C, Mendelsohn LG, Seitz DE and Tonkinson JL (1999) Cellular pharmacology of MTA: a correlation of MTA-induced cellular toxicity and in vitro enzyme inhibition with its effect on intracellular folate and nucleoside triphosphate pools in CCRF-CEM cells. *Semin Oncol* **26**: 48–54

Clavel M, Vermorken JB, Cognetti F, Capperaere P, de Mulder PH, Schornagel JH, Tueni EA, Verweij J, Wildiers J, Clerico M et al (1994) Randomized comparison of cisplatin, methotrexate, bleomycin and vincristine (CABO) versus cisplatin and 5-fluorouracil (CF) versus cisplatin (C) in recurrent or metastatic squamous cell carcinoma of the head and neck. A phase III study of the EORTC Head and Neck Cancer Cooperative Group. *Ann Oncol* **5**: 521–526

Dimery IW and Hong WK (1993) Overview of combined modality therapies for head and neck cancer. *J Natl Cancer Inst* **85**: 95–111

Fleming ID, Cooper JS, Henson DE et al (1997) AJJC Cancer Staging Manual, 5th ed.. In: *American Joint Committee on Cancer* editors. Lippincott-Raven Publishers: Philadelphia, PA

Forastiere AA, Metch B, Schuller DE, Ensley JF, Hutchins LF, Trizzi P, Kish JA, McClure S, Van Feldt E, Williamson SK et al (1992) Randomized comparison of cisplatin plus fluorouracil and carboplatin plus fluorouracil versus methotrexate in advanced squamous-cell carcinoma of the head and neck: a Southwest Oncology Group study. *J Clin Oncol* **10**: 1245–1251

Jacobs C, Meyers F, Hendrickson C, Kohler M and Carter S (1983) A randomized phase III study of cisplatin with or without methotrexate for recurrent squamous cell carcinoma of the head and neck. A Northern California Oncology Group study. *Cancer* **52**: 1563–1569

John W, Picus J, Blanke C, Clark J, Shulman LN, Thorton D, Rowinsky E and Loehrer PJ Sr (2000) Activity of multitargeted antifolate (pemetrexed disodium, LY231514) in patients with advanced colorectal carcinoma: results

 *© 2001 Cancer Research Campaign*

BJOC 01-2010 649-655 20/8/01 3:57 pm Page 655

from a phase II study. *Cancer* **88**: 1807–1813
Landis SH, Murray T, Bolden S and Wingo PA (1999) Cancer statistics, 1999. *CA Cancer J Clin* **49**: 8–31
Mandema J, Verotta D and Sheiner L (1992) Building population pharmacokinetic-pharmacodynamic models. I. Models for covariate effects. *J. Pharmacokin. Biopharm* **20**: 5111–5128
Mendelsohn LG, Gates SB, Habeck LL, Shackelford KA, Worzalla J, Shih C and Grindey GB (1996) The role of dietary folate in modulation of folate receptor expression, folylpolyglutamate synthetase activity and the efficacy and toxicity of lometrexol. *Adv Enzyme Regul* **36**: 365–381
Morgan SL, Baggott JE, Vaughn WH, Young PK, Austin JV, Krumdieck CL and Alarcon GS (1990) The effect of folic acid supplementation on the toxicity of low-dose methotrexate in patients with rheumatoid arthritis. *Arthritis Rheum* **33**: 9–18
Niyikiza C, Baker SD, Johnson R, Walling J, Thornton D, Seitz D and Allen R (1998) MTA (LY231514): relationship of vitamin metabolite profile, drug exposure, and other patient characteristics to toxicity. *Ann Oncol* **9** (Suppl 4): 609P (Abstr)
O'Dwyer PJ, Nelson K and Thornton DE (1999) Overview of phase II trials of MTA in solid tumors. *Semin Oncol* **26**: 99–104
Postmus PE and Green MR (1999) Overview of MTA in the treatment of non-small cell lung cancer. *Semin Oncol* **26**: 31–36
Pivot X, Niyikiza C, Poissonnet G, Dassonville O, Bensadoun RJ, Thyss A, Demard F, Kayitalire L and Schneider M (2000) Multivariate analysis of prognostic factors in local recurrent and metastatic head and neck cancer: implication for clinical trials. *Proc Am Soc Clin Oncol* **19**: 414 (Abst 1634)
Recondo G, Armand JP, Tellez-Bernal E, Domenge C, Nelehradek M, De Vathaire F, Wibault P, Richard JM and Cvitkovic E (1991) Recurrent and/or metastatic head and neck squamous cell carcinoma: a clinical, univariate and multivariate analysis of response and survival with cisplatin-based chemotherapy. *Laryngoscope* **101**: 494–501
Rinaldi DA, Kuhn JG, Burris HA, Dorr FA, Rodriguez G, Eckhardt SG, Jones S, Woodworth JR, Baker S, Langley C, Mascorro D, Abrahams T and Von Hoff DD (1999) A phase I evaluation of multitargeted antifolate (MTA, LY231514), administered every 21 days, utilizing the modified continual reassessment method for dose escalation. *Cancer Chemother Pharmacol* **44**: 372–380
Rusthoven J, Eisenhauer E, Butts C, Gregg R, Dancey J, Fisher B and Iglesias J (1999) Multitargeted antifolate LY231514 as first-line chemotherapy for patients with advanced non-small-cell lung cancer: a phase II study. *J Clin Oncol* **17**: 1194–1199
Schornagel JH, Verweij J, de Mulder PH, Cognetti F, Vermorken JB, Cappelaere P Armand JP, Wildiers J, de Graff A, Clavel M et al (1995) Randomized phase III trial of edatrexate versus methotrexate in patients with metastatic and/or recurrent squamous cell carcinoma of the head and neck: a European Organization for Research and Treatment of Cancer Head and Neck Cancer Cooperative Group study. *J Clin Oncol* **13**: 1649–1655
Shih C, Chen VJ, Gossett LS, Gates SB, MacKellar WC, Habeck LL, Shackelford KA, Mendelsohn LG, Soose DT, Patel VF, Andis SL, Bewley JR, Rayl EA, Moroson BA, Beardsley GP, Kohler W, Ratnam M and Schultz RM (1997) LY231514, a pyrrolo[2,3-d]pyrimidine-based antifolate that inhibits multiple folate-requiring enzymes. *Cancer Res* **57**: 1116–1123
Smith GK, Amyx H, Boytos CM, Duch DS, Ferone R and Wilson HR (1995) Enhanced antitumor activity for the thymidylate synthase inhibitor 1843U89 through decreased host toxicity with oral folic acid. *Cancer Res* **55**: 6117–6125
Vogl SE, Schoenfeld DA, Kaplan BH, Lerner HJ, Engstrom PF and Horton J (1985) A randomized prospective comparison of methotrexate with a combination of methotrexate, bleomycin, and cisplatin in head and neck cancer. *Cancer* **56**: 432–442
Vokes EE, Weichselbaum RR, Lippman SM and Hong WK (1993) Head and neck cancer [see comments]. *N Engl J Med* **328**: 184–194
World Health Organization (2000) The World Health Report 2000. Health Systems: Improving Performance. http://www.who.int/whr

*© 2001 Cancer Research Campaign* 
